# *Rapper***tk**: a versatile engine for discrete restraint-based conformational sampling of macromolecules

**DOI:** 10.1186/1472-6807-7-13

**Published:** 2007-03-21

**Authors:** Swanand P Gore, Anjum M Karmali, Tom L Blundell

**Affiliations:** 1 Department of Biochemistry, University of Cambridge, Tennis Court Road, Cambridge CB2 1GA UK

## Abstract

**Background:**

Macromolecular structures are modeled by conformational optimization within experimental and knowledge-based restraints. Discrete restraint-based sampling generates high-quality structures within these restraints and facilitates further refinement in a continuous all-atom energy landscape. This approach has been used successfully for protein loop modeling, comparative modeling and electron density fitting in X-ray crystallography.

**Results:**

Here we present a software toolkit (*Rapper***tk**) which generalizes discrete restraint-based sampling for use in structural biology. Modular design and multi-layered architecture enables *Rapper***tk **to sample conformations of any macromolecule at many levels of detail and within a variety of experimental restraints. Performance against a *C*_*α*_-tracing benchmark shows that the efficiency has not suffered despite the overhead required by this flexibility. We demonstrate the toolkit's capabilities by building high-quality *β*-sheets and by introducing restraint-driven sampling. RNA sampling is demonstrated by rebuilding a protein-RNA interface. Ability to construct arbitrary ligands is used in sampling protein-ligand interfaces within electron density. Finally, secondary structure and shape information derived from EM are combined to generate multiple conformations of a protein consistent with the observed density.

**Conclusion:**

Through its modular design and ease of use, *Rapper***tk **enables exploration of a wide variety of interesting avenues in structural biology. This toolkit, with illustrative examples, is freely available to academic users from .

## 1 Background

Atomic structures of biological macromolecules give key insights into their biochemical function and are determined by conformational optimization within the landscape defined by experimentally derived and knowledge-based restraints. Molecular dynamics and minimization, implemented in popular softwares like Charmm [1] and Gromacs [2], play a significant role in this process. However, all-atom forcefields used by these methods give rise to complex and rugged energy landscapes, which often create substantial difficulties in locating meaningful minima. This task can be facilitated if seed conformations are obtained within convergence radii of these minima.

Recent studies have illustrated this for proteins in various contexts [3]. Analyses of high resolution structures have yielded discrete preferred conformational states for protein backbones and sidechains [4-7]. Protein loops modeled with weighted sampling of knowledge-based preferences, excluded volume restraints and ideal stereochemistry, when further optimized with an all-atom forcefield, have accurately predicted the native conformation [8,9]. The combination of knowledge-based local preferences (for fragments smaller than 10 residues) with non-local physical energy terms like hydrophobic burial and hydrogen bonding in a simulated annealing protocol has been effective in protein structure prediction [10], homology modeling [11] and structure determination [12]. Interpretations of crystallographic data of both high [13] and low [14] resolutions have been achieved by combining discrete and continuous approaches.

**Figure 1:**
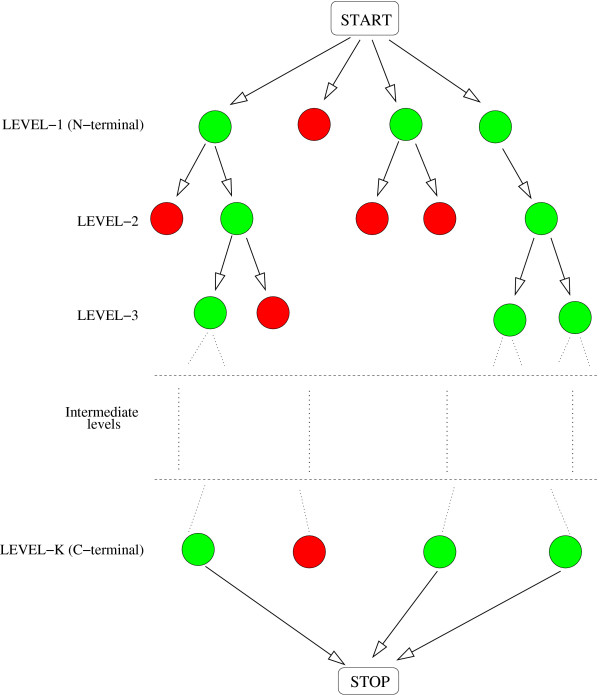
The central search algorithm in Rapper is a blend of genetic algorithm and branch-and-bound approach (GABB). Red nodes represent restraints-violating conformation extensions and green nodes stand for the restraints-obeying ones. Some conformational extensions may be left unsampled (not shown). Subtrees emanating from green nodes only are explored further. Set of green nodes at each level is kept below a fixed size (population size), and this allows conformational exploration in time proportional to protein length, leading to an ensemble of restraints-satisfying conformations.

The promise of this hybrid approach has not yet been fully exploited; for instance it has not been used to assess conformational ensembles to enhance structure determination with NMR and EM data, to explore flexibility of ligands including macromolecules such as RNA or to examine diversity at macromolecular interfaces. Our approach, encoded in RAPPER [15] (Fig. 1), has been applied succesfully to a range of protein modeling problems where restraints have been introduced from knowledge of structures or experimental obsvservations. But RAPPER is limited in applicability due to its inflexibility in molecular representation (proteins only), sampling direction (N to C) and search algorithm (Genetic Algorithm with Branch and Bound : GABB). These limitations have to be removed if the idea of discrete restraints-based sampling is to be applied to new problems. We found that this was quite challenging within the RAPPER codebase (> 30,000 lines of C++ code).

In this paper we describe an alternative framework, *Rapper***tk**, which (a) programmatically decouples the logically distinct concepts like search algorithms, knowledge-based confomational preferences, sampling and building techniques and (b) provides access to them with a scripting language. The former reduces development time by allowing modules to be treated in isolation – e.g. RNA sampling and building can be implemented independent of GABB. The latter speeds up the process of adapting the software to new scenarios, say by coding high level tasks like parsing and file manipulations in the scripting language. We show that both impact scientific productivity by allowing faster application of discrete restraints-based sampling to new problems. Analogous to MD softwares which provide a platform to run MD/minimization schedules, *Rapper***tk **provides a platform for discrete restraints-based sampling and reproduces RAPPER functionality for proteins as a special case. Following sections describe the design, implementation and benchmarking of *Rapper***tk**. We demonstrate that *Rapper***tk **has a flexible, robust and easy-to-use software library which generalizes and builds upon the major concepts from RAPPER methodology in a modular, multi-layered fashion.

## 2 Implementation

**Figure 2:**
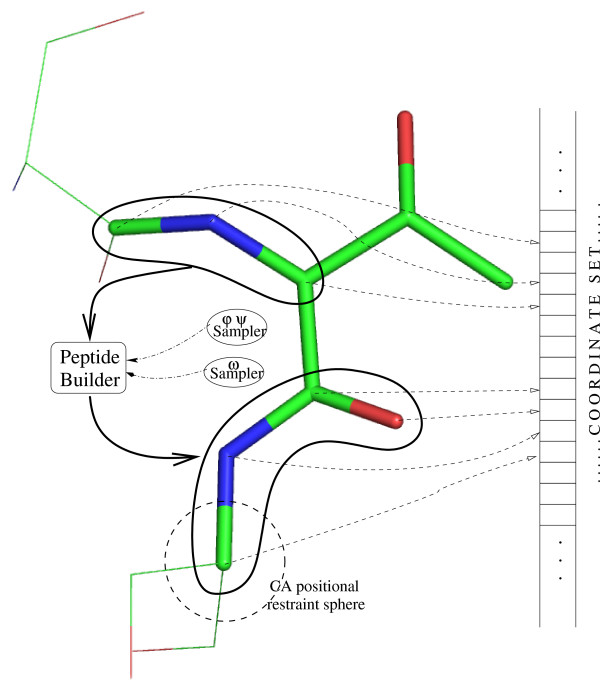
Basic concepts in *Rapper***tk**. A sampler samples discrete conformational preferences (e.g. *ϕ*, *ψ*, *ω*). A builder uses the sample and calculates a set of unknown coordinates from a set of known coordinates (e.g. peptide building). A restraint checks that the calculated coordinates satisfy some geometric criterion (e.g. whether calculated *C*_*α *_coordinate lies within a spherical region).

**Figure 3:**
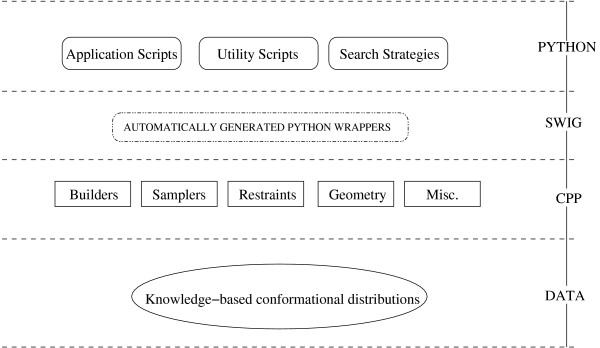
*Rapper***tk **architecture – modular and layered. Modules written in C++ are exposed to PYTHON using automatic wrappers generated with SWIG.

Fig. 2 shows a typical step in RAPPER-like incremental sampling. This involves three distinct steps : sampling of dihedral angles *ϕ*, *ψ*, *ω*, building coordinates for the next peptide using those of the previous and checking the *C*_*α*_-positional restraint. This suggests the concepts of sampler, builder and restraint. RAPPER maintains a population of conformers and executes these steps repeatedly on them according to GABB. This can be abstracted as search strategy which is responsible for correct ordering and execution of samplers, builders and restraints. In the modular, layered design of *Rapper***tk **(Fig. 3), application scripts reside at a level higher than search strategies – they carry out the task of preprocessing, creating necessary builders, samplers and restraints for the problem at hand, and passing them to the appropriate strategy.

We have chosen a C++/SWIG/PYTHON style of coding, whereby the interface of C++ code is exposed in PYTHON by generating suitable wrappers automatically with SWIG. Such architecture has become popular among academic softwares (e.g. Xplor-NIH [16]) as it provides robustness without losing the fluidity needed in academic implementations. We now describe the major concepts in more detail.

### 2.1 Coordinates

Different sets of coordinates need to be maintained in order to allow for sets of conformers, either for population-based searches or for using ensemble averaged restraints. Some coordinates are known and fixed, e.g. secondary structure elements in a loop building exercise. Each point has an associated hard-sphere (van der Waals) radius adapted from those used in PROBE [17]. A high-level application script generates the coordinates. Builders and restraints operate upon specified indices in given coordinates.

### 2.2 Samplers

A sampler chooses a datum from an underlying distribution of conformational preferences by random weighted sampling. Well-known examples are weighted *ϕ*, *ψ *sampling for protein backbone [18], RNA backbone [4] and sidechain rotamer sampling [6], all derived from high quality crystallographic structures. New types of sampling can be easily incorporated by writing a new sampler for the corresponding builder, say tri-*ϕ*, *ψ *sampler for tripeptide fragments, substructure sampler etc.

### 2.3 Restraints

Values of various geometric entities are useful in constraining the conformational space, e.g. internuclear distances derived from NMR NOEs, electron-density from X-ray analyses, *C*_*α *_positional information from templates in comparative modelling, and so on. A restraint object holds the information of points on which it is to be tested, and the method of testing. A restraint is generally binary; it is either satisfied or violated. A restraint can be also be optional, i.e. it can be discarded if sampling consistently fails due to that restraint.

### 2.4 Builders and followers

A builder consists of the indices of coordinates it uses and those it calculates, along with the calculation technique. For instance, the *ϕ*, *ψ*-based peptide backbone builder uses coordinates of {*C*^*i*-2^, *N*^*i*-1^, *C*_*α*_^*i*-1^} to calculate coordinates of {*C*^*i*-1^, *N*^*i*^, *C*_*α*_^*i*^}; {*C*^*i*-1^, *N*^*i*^, *C*_*α*_^*i*^, *C*_*β*_^*i*^, *C*^*i*^, *N*^*i*+1^} coordinates are used by a backbone-dependent sidechain builder; and so on. Thus builder is an abstraction of coordinate calculations operating on input and output indices within a coordinate set. A builder may have an empty input set or may have only known coordinates as inputs, in which case it is called a *seed *builder (e.g. peptide N terminal anchor builder). There is a maximum number of trials a builder can undertake to extend a conformation; this will depend upon the conformational space available to sample. In order to avoid futile sampling, the builder may implement a session in which only unique samples are used, thus improving sampling diversity. Follower is a concept specific to population-based searches. A builder is another's follower if it is advisable to execute it in the same population-search step as the leading builder. This was an improvisation first used during *C*_*α*_-trace scripting to build sidechain immediately after the relevant mainchain.

### 2.5 Sampling Strategy

The sampling strategy orchestrates the builders and restraints systematically to generate conformations. The sampling strategy can be divided into ordering and execution of restraints and builders. Automatic ordering allows the application script to create builders and restraints in any convenient order. Because strategies are coded in PYTHON, it is easy to write a new strategy.

#### 2.5.1 Ordering of builders and restraints

**A **correct strategy must calculate the order of execution for builders and restraints. There is a partial ordering induced on builders due to their input and output coordinates, i.e. a builder may not be executed unless its input coordinates have been computed, except for seed builders. Thus there is a digraph of builders, with possibly many seed builders and others depending on one or more builders. Restraints can be checked only after all coordinates to be tested have been computed, hence there is restraint-builder dependence. An efficient strategy must test a restraint as early as possible in order to avoid sampling the disallowed conformational space. Once a builder succeeds or fails in its task, an efficient sampling strategy must use the builder dependence digraph to identify the builder to be attempted next. The strategy currently implemented in *Rapper***tk **determines the builder order by topologically sorting the builder digraph, more specifically as follows:

• In case of multiple seed (parentless) builders, a dummy builder is assumed to be their parent. A procedure similar to DFS (depth first search) is used to assign unique parents to all nodes, i.e. convert the digraph into a tree. A node appears as child of another node only if the latter is the only unvisited parent of the former.

• The size of subtree rooted at each node is found.

• Using DFS again, an order is established for the nodes. When a node is popped off the DFS stack, its children are pushed onto the stack in the ascending order of subtree sizes.

• The order thus obtained is the final ordering used by the default strategy. If a builder fails, its unique parent builder may be executed, and the results of the parent and all its children discarded. If a builder succeeds, the builder next in order may be called.

• From this builder order, restraints are identified for each builder such that they have all the necessary points computed after the builder. Thus every builder has associated restraints to check after it is executed.

**Figure 4:**
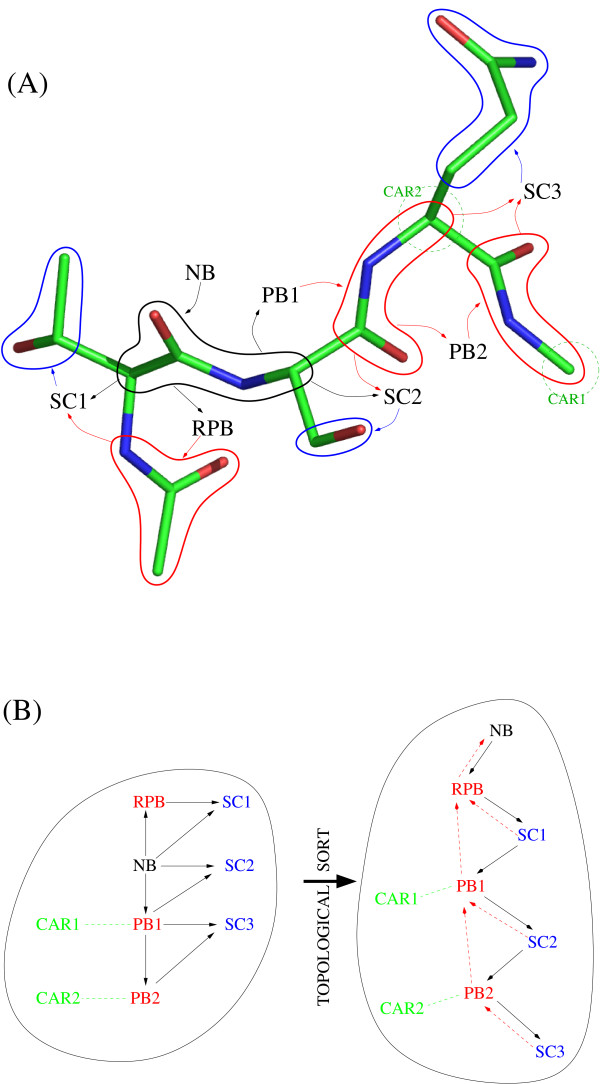
Automatic ordering of builders and restraints involved in sampling a three-residue peptide. (A) shows the builders and their output coordinates, *C*_*α *_positional restraints and the coordinates they test. NB (N-anchor-builder), PB (*ϕ*, *ψ*-sampling based peptide builder), RPB (PB-like, but in reverse direction), SC (backbone dependent sidechain builder) are used. A builder points into the coordinates it computes and is pointed at by the output coordinates of the builders it depends on. Restraints contain the coordinates they test. (B) On left is the dependence digraph on builders and restraints in (A). A builder node depends upon the builder node it is pointed from. A restraint node is connected to a builder after execution of which it is possible to execute the restraint. Topological sort (as described in text) on this digraph results (on right) in a linear forward order (black) and fallback order (red) on builders.

As an illustration, consider conformational sampling of a three residue peptide (see Fig. 4) under the *C*_*α *_spherical positional restraints. Four kinds of builders are employed. NanchorBuilder uses the first two *C*_*α *_restraints to anchor the peptide. Backbone-dependent sidechain builder is used for sampling sidechains. Since this builder requires parts of the backbone from adjacent residues also, two dummy Gly residues are added, one each at the beginning and end of the tripeptide. PeptideBuilder is used to build peptides in forward and backward directions. NanchorBuilder is the seed builder as it has no input points. Reverse PeptideBuilder, PeptideBuilder-1 and SidechainBuilder-1 depend on it because their input coordinates are partly or completely contained in its output coordinates. Similarly, SidechainBuilder-3 depends upon PeptideBuilder-1 and PeptideBuilder-2. Restraints CARestraint-1 and 2 depend upon PeptideBuilder-1 and 2. From these dependences, a directed graph can be constructed with builders and restraints as nodes. Topological sort on this graph produces a linear order of the builders, which suggests the builder to be tried after a successful (restraints-satisfying) builder. The backward ordering (or *fallback ordering*) determines the builder to be called after an unsuccessful (not satisfying restraints) builder.

#### 2.5.2 Execution of builders and restraints

Once the ordering among builders and restraints is established, various search strategies can be used to sample conformational space. The simplest is an exhaustive search, where each restraints-satisfying option available to a builder is explored. RAPPER uses PopulationSearch algorithm (GABB) as mentioned earlier. GABB limits the number of restraints to be checked at every extension step and provides a pool of fit parents to build upon. Each parent is allowed to contribute more than one child and parents compete to put their children in the children pool. In addition to PopulationStrategy, *Rapper***tk **provides a minor variation which allows limited backtracking (using fallbacks described earlier). The number and size of backtrack steps can be specified. In cases where the parents are not extensible at a certain step, the population search is restarted some steps earlier, determined by number and size of backtrack step specified. This saves the cost of starting from first step in case of failure at an advanced step.

### 2.6 Spatial grid for checking clashes

Steric clashes are a very important restraint on conformational freedom. Hence the output of every builder is verified with a 3D grid that uses geometric caching to check the clashes efficiently. A GridHelper is provided to the grid to modify clash-checking functionality according to the application requirements. For instance, in atomic models, first and second covalent neighbours of an atom need not be clash-checked, the van der Waals radius of sidechain atoms needs to be reduced due to discrete sidechain rotameric states, etc.

### 2.7 PDB reader, model renderer etc

The i/o functionality is written in PYTHON. PDB reader is largely adapted from a previous work [19]. ModelRenderer is currently a PDB writer, but can be extended to write models in other formats too. ModelRenderer is invoked by the strategy when it succeeds in sampling a conformation within given restraints.

### 2.8 Application scripts

Application scripts are high-level PYTHON scripts which generate problem-specific context by preprocessing given information and creating necessary *Rapper***tk **components to be used by the search strategy. They can be invoked as execution modes from *Rapper***tk **launcher script. Application scripts are assisted by various utility scripts like the one for creating a standard set of builders and restraints.

## 3 Results

### 3.1 Benchmarking

As tracing a polypeptide chain is central to all the tasks performed by RAPPER, we compare *Rapper***tk**'s performance at chain tracing with that of RAPPER for 9 large (> 300 residues) proteins from the [20] benchmark set (see Table 1). Ensembles of 50 models were generated and the average RMSD values for the ensembles calculated. If the conformational search could not generate a model within 24 hours of computational time, the search was considered unsuccessful. Mainchain-only models were generated using *C*_*α *_restraint radii of 0.5, 1, 1.5 and 2Å. The *C*_*α *_restraint threshold defines the radius of the sphere within which the *C*_*α *_atom of the modelled residue is restrained to lie. The centre of this sphere is given by the native *C*_*α *_position. All-atom models were generated under *C*_*α *_restraint of 1Å and 2Å restraint on the centroid of the sidechain atoms. The van der Waals radii were reduced by 25% to compensate for the fact that only specific sidechain rotamers were allowed. Sidechain centroid restraint places and orients the side chain atoms with respect to the mainchains and affects bulky side chains more than the smaller sidechain groups.

**Figure 5:**
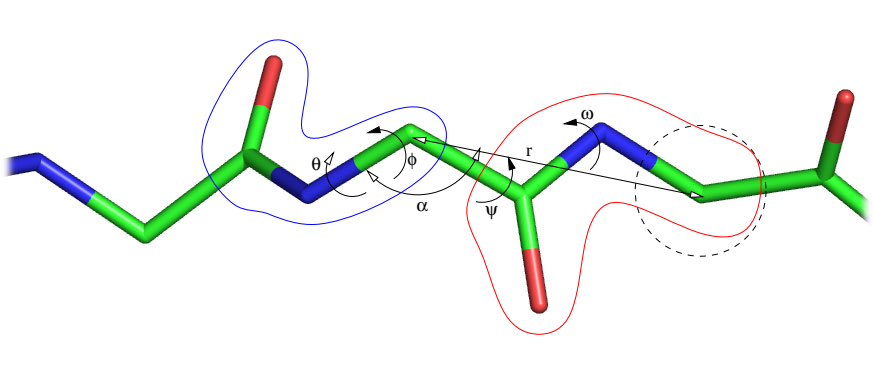
Guided sampling. Location of *C*_*α*_^*i *^can be described by specifying locations of *C*^*i*-2^, *N*^*i*-1^, *C*_*α*_^*i*-1 ^along with {*r*, *α*, *θ*} or {*ϕ*, *ψ*, *ω*}. This leads to one-to-many mapping between {*r*, *α*, *θ*} and {*ϕ*, *ψ*, *ω*}.

*Rapper***tk **can trace either from N to C terminal (forward) or in the C to N (backward) directions, with and without sidechains, in guided or standard sampling modes. Standard sampling is RAPPER-like *ϕ*, *ψ *sampling which is unaware of the *C*_*α *_restraint to be satisfied. Such sampling can be the bottleneck when restraints are tight or only a small portion of the restraint spheres are reachable geometrically. Hence we have also incorporated *guided *sampling in which the sampler is aware of the restraint and produces samples within that restraint. As shown in Fig. 5, the location of *C*_*α*_^*i *^is defined by *C*^*i*-2^, *N*^*i*-1^, *C*_*α*_^*i*-1 ^and *r *(distance between *C*_*α*_^*i*-1^, *C*_*α*_^*i*^), *α *(angle *N*^*i*-1 ^-*C*_*α*_^*i*-1 ^-*C*_*α*_^*i*^), *θ *(torsion angle *C*^*i*-2 ^-*N*^*i*-1 ^-*C*_*α*_^*i*-1 ^-*C*_*α*_^*i*^). Thus the restraint sphere is sampled spatially by the guided sampler to obtain *r*, *α*, *θ*, samples. Corresponding *ϕ*, *ψ*, *ω *values are found using a pre-calculated mapping from *r*, *α*, *θ *to allowed *ϕ*, *ψ*, *ω*. Since this mapping is one-to-many, a random sample is taken from available *ϕ*, *ψ*, *ω *values. Such sampling ensures that the restraint sphere is sampled efficiently while still using *ϕ*, *ψ *values from the allowed region of Ramachandran plot.

#### 3.1.1 Mainchain modelling

In addition to comparing the main chain modelling accuracy between RAPPER and *Rapper***tk **in standard forward mode, models were built in the backward (C to N) mode in order to check whether the performance varies. Table 2 shows the model accuracy under a spherical positional restraint of radius 1Å on *C*_*α *_atoms. Similar values of mainchain and *C*_*α *_RMSDs obtained demonstrate that performance of *Rapper***tk **is comparable to that of RAPPER and consistent across the whole target set. The low standard deviation values within each ensemble show that all the three approaches produce tight clusters containing models that are all equally acceptable. Larger restraint radii result in looser restraints and give models that deviate further from the native structures. RMSD values in Table 3 demonstrate that both RAPPER and *Rapper***tk **perform equally well under different *C*_*α *_restraint thresholds. For the restraint radius of 0.5Å both RAPPER and *Rapper***tk **failed to find complete ensembles for proteins 4enl, 8abp and 8tln. For 8tln, the conformational search repeatedly failed at Leu-133. Since the conformational search builds one residue at a time, slight errors introduced earlier can sometimes make it difficult to find a suitable conformation for a residue causing repeated failures at the same position. This limitation can be circumvented by building the peptide chain in the reverse direction. Using backward building for 8tln, 5 models could be found having an average main chain RMSD of 0.4lÅ (0.01) and a *C*_*α *_RMSD of 0.35Å (0.01). Models for proteins 8abp and 4enl were built using the guided sampling mode in *Rapper***tk**.

#### 3.1.2 All atom modelling

As can be seen from Table 4, the model accuracies for RAPPER and *Rapper***tk **are comparable and do not vary significantly across the different proteins. The average all atom RMSD for RAPPER and *Rapper***tk **for the entire protein set is 1.07Å and 1.08Å respectively. On using the guided sampling mode within *Rapper***tk **there is a 0.06Å to 0.08Å reduction in the main chain and a 0.07Å to 0.09Å reduction in the all atom RMSD values. The average all atom RMSD over the target set also reduces to l.0Å. The average *χ*_1 _error is comparable for RAPPER (42.3°), *Rapper***tk **(42.0°) and guided sampling (41.6°). The average *χ*^1,2 ^values for the three approaches are also similar, 42.4° for RAPPER, 42.4° for *Rapper***tk **and 42.1° for *Rapper***tk **using guided sampling.

#### 3.1.3 Computational Cost and Quality Check

**Figure 6:**
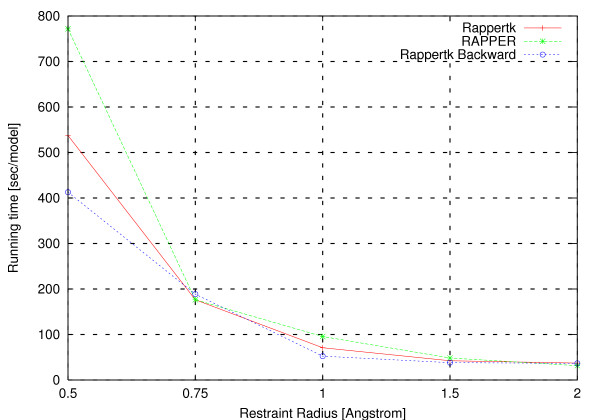
Computational cost scales as a function of *C*_*α *_restraint radius for RAPPER (squares), *Rapper***tk **(diamonds) and *Rapper***tk **using backward building (triangles). 5cpa is exluded.

**Figure 7:**
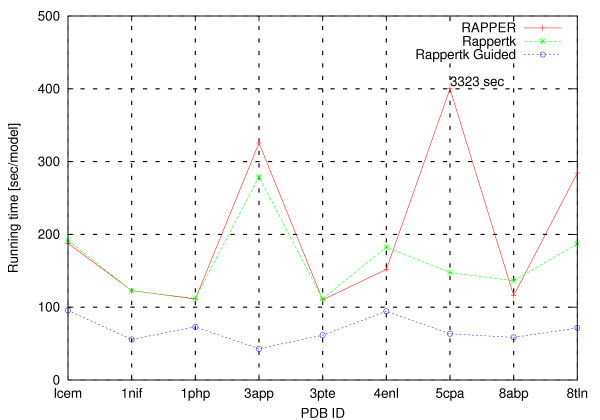
Computational cost for all-atom modelling across target set. The average time required to build a successful model is shown for RAPPER (diamonds), *Rapper***tk **(squares) and *Rapper***tk **with guided sampling (triangles).

Model quality was assessed using PROCHECK [21]. All structures have main chain bond lengths and angles within the limits of the standard deviation of their small molecule values and also have good sidechain stereochemistries. Computational cost scales with the size of the restraint sphere used to generate the models. As can be seen from Fig. 6 the computational cost for *Rapper***tk **is less than that for RAPPER for restraint radii 0.5, 1, and 1.5 l.5Å. The average time taken by *Rapper***tk **under a *C*_*α *_restraint of 2Å is only slightly higher at 37 s/models compared to RAPPER which takes 32 s/model. There is a visible improvement in speed at the restraint radius of 0.5Å. This demonstrates that *Rapper***tk **is more able to find a solution within a very tight restraint network with fewer failed attempts. Fig. 7 shows the computational cost for all-atom modelling of each protein in the target set. The time taken to build a successful all atom model by RAPPER is similar to that taken by *Rapper***tk**. For 5cpa, RAPPER repeatedly failed at TYR:198 which has an unusual *ω *angle of 154.5°. RAPPER takes an average of 3323 s to find a solution whereas *Rapper***tk **is able to build a model in an average time period of 147 s. On using guided sampling the computational cost siginificantly decreases. The average time taken reduces to 69 s/model compared to the average time of 165 s/model taken by *Rapper***tk **and 176 s/model by RAPPER. Also on using guided sampling, the cost is nearly the same across the set, irrespective of the stereochemistry of the individual structures.

### 3.2 Illustrations

We now describe the use of *Rapper***tk **to carry out some new sampling tasks.

#### 3.2.1 Protein-ligand interface sampling in electron density

Protein ligand interactions are central to understanding the roles of ligands as well as the mechanisms of enzymes. The approximate location of a ligand is often known but small ligands often have poor electron density. This scenario is suitable for automatically fitting various ligand conformations into the density with *Rapper***tk**, thus creating an ensemble for further refinement. From a recent paper on automatic modeling of ligands [22], we chose a medium resolution (2.6Å) structure (1di9) of p38 kinase in complex with a quinazoline ligand.

In order to describe the degrees of freedom in a ligand, a file format was devised. It describes the ligand's bootstrapping (init lines), rotatable bonds (rotbond lines) and internal distance restraints (mindist lines). Builders and restraints are created using the information given in this file. Covalent bond lengths and angles are not altered from the initial coordinates given as input.

**Figure 8:**
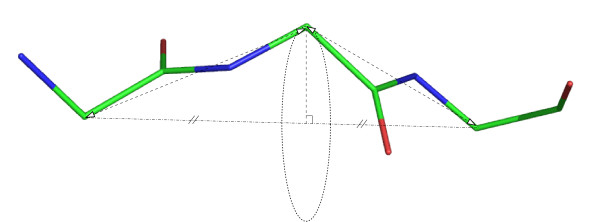
Loop closure procedure in *Rapper***tk**. *C*_*α*_^*i *^lies on a circle centered at the mid-point of line joining *C*_*α*_^*i*-1 ^and *C*_*α*_^*i*+1^. Radius of the circle is determined by length of the line. The circle is in a plane perpendicular to the line. Candidate *C*_*α*_^*i *^positions are sampled on this circle and {*r*, *α*, *θ*} – {*ϕ*, *ψ*, *ω*} mapping (explained earlier in relation to guided sampling) is used to select a position.

Depending on ligand proximity, small sections of protein chains are identified and sampled using a loop sampling and closure procedure. Loop closure samples the location of *C*_*α*_^*i *^given the locations of *C*_*α*_^*i*-1 ^and *C*_*α*_^*i*+1 ^as shown in Fig. 8. Sidechain centroid restraint and *C*_*α *_restraint are lenient close to ligand.

Electron density restraints are employed using the excellent Clipper libraries [23] for crystallographic computations. The deposited PDB structure is used to phase the structure factor amplitudes and to obtain an electron density map. EDrestraint is satisfied by builder outputs which lie in reasonable density (> 0.25 *σ*) and have good mean density (> 1 *σ*). EDrestraints are optional except for the ligand. EDrestraints operate on the output of each builder.

**Figure 9:**
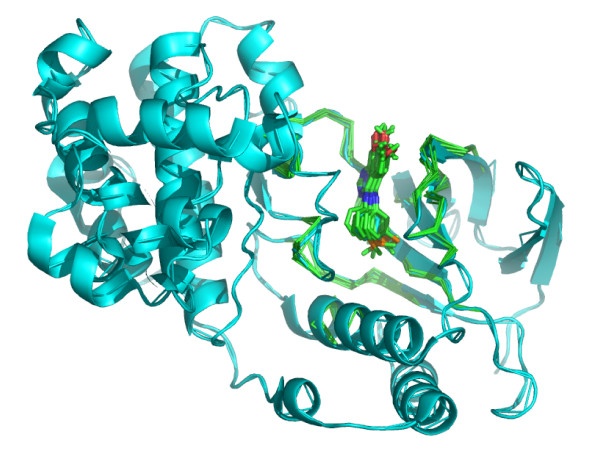
p38 kinase in complex with a quinazoline ligand (PDB 1di9). Green conformations are generated by *Rapper***tk**. Sticks show the ligand. Electron density not shown for clarity.

This scheme of flexible-protein flexible-ligand yields an ensemble of protein-ligand interface conformations which are consistent with the expected degrees of freedom of ligand, electron density, hard-sphere clash restraints and covalent geometry of the protein (Fig. 9). Further refinement and ensemble interpretation will be addressed in future work. Apart from crystallographic application, such sampling can be used by small molecule docking programs also to generate trial conformers of the ligand and protein.

#### 3.2.2 Protein-RNA interface sampling

**Figure 10:**
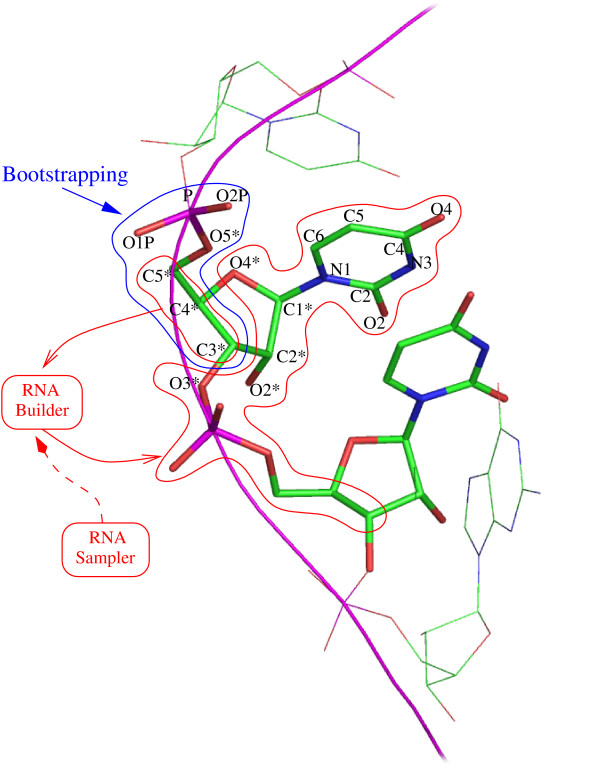
*Rapper***tk **procedure for RNA sampling. Bootstrap builder assigns the location of a few initial atoms of the first nucleotide by rigid-body transformation from given structure satisfying the positional restraints specified on C5*, C4*, C3* atoms. Chain extension is carried out by RNAbuilder according to the backbone dihedrals sampled by RNAsampler (using the rotamericity described in [4]). RNAbuilder builds the corresponding sugar and base also.

Although RNA conformational preferences are harder to identify due to the much larger conformational space (7 backbone dihedral angles), recent analysis has revealed the ro-tameric nature of the RNA backbone [4]. We use these preferences to extend the RNA chain as shown in Fig. 10. Bootstrapping copies the initial few atoms from the given structure to the region specified by restraints on them. Incremental build of the RNA chain is done by RNAsuiteBuilder, which depends on atoms {C5*, C4*, C3*} and builds atoms {O3*, P, O1P, O2P, O5*, C5*, C4*, C3*} along with sugar and base.

**Figure 11:**
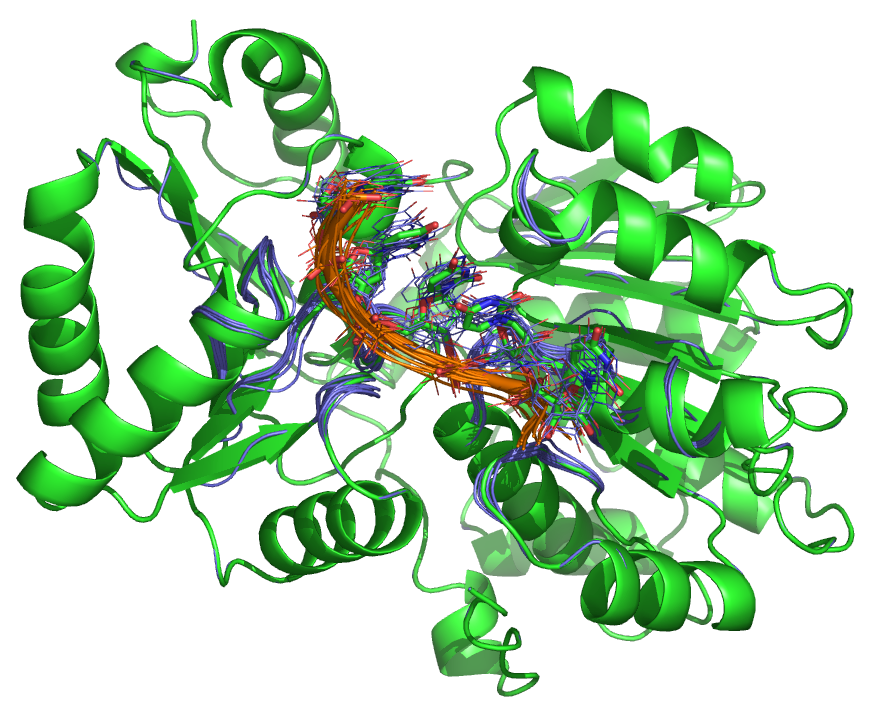
Multiple conformations possible for a protein-RNA interface (helicase-core region of Vasa, chains A, E in PDB 2db3) within electron density restraints. Native structure is rendered as sticks and cartoon, five models as lines and ribbon. Electron density not shown for clarity.

In this illustration (Fig. 11), we choose protein chain A and RNA chain E from a recently solved protein-RNA complex (helicase-core region of Vasa bound to a single stranded RNA [24]). We identify sections of protein chain in close proximity to the RNA. These sections are later sampled as loops with loop closure and restrained with *C*_*α *_and sidechain centroid positional restraints. RNA bootstrap builder regards {C5*, C4*, C3*, P, O1P, O2P, O5*} atoms of the first nucleotide as a rigid body and translates/rotates it so that C5*, C4*, C3* atoms are within 2Å of native positions. During incremental building, the C3* atom is restrained to lie within 2Å of the native C3* atom. As before, the deposited PDB structure is used to phase the deposited structure factor amplitudes and builders are restrained to build within a mean electron density of 1 *σ *.

Generation of multiple conformations of protein-RNA interface with *Rapper***tk **can be useful in deriving multiple interpretations permitted by the crystallographic data. Interface diversity thus assessed may lead to novel insights into function. This issue will be addressed in detail in a future study.

#### 3.2.3 Sampling *β *sheets

**Figure 12:**
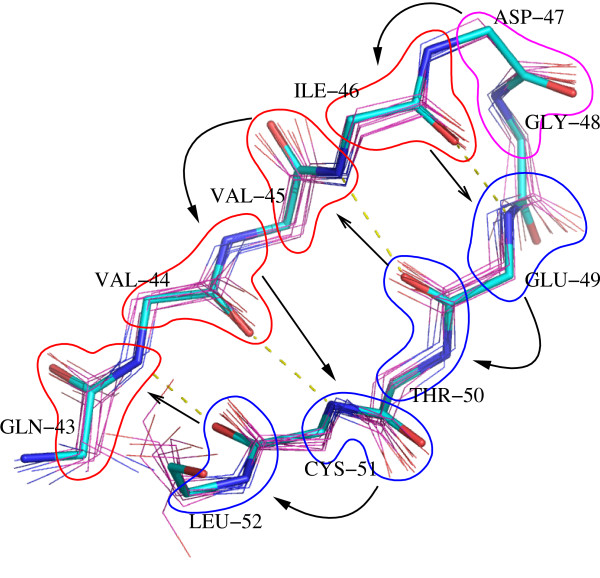
*β*-hairpin building. Blue is NanchorBuilder's output, red is that of forward PeptideBuilder, brown is that of backward PeptideBuilder and magenta that of Peptide-BridgeBuilder. Dotted lines show the distance restraints used for hydrogen bonding in addition to 0.5Å *C*_*α *_positional restraints.

In low-resolution crystallographic or EM data, salient features of the structure (*β*-sheet or *α*-helix) are more detectable than the terminal regions or loops, making it desirable to start building a model at such features. *α*-helices are easier to sample than *β*-sheets because hydrogen bond restraints in helices are sequential unlike those in sheets. Hence sequential sampling is inefficient for the later strands in a sheet. As *Rapper***tk **is not restricted to sequential sampling, a *β*-hairpin can be built as shown in Fig. 12, by bootstrapping at the linker of the strands and extending in forward and reverse directions. The building order is zigzag and helps in maintaining strict hydrogen bond geometry (distance O-N within between 1.5Å, 3.5Å angle C-O-N > 100°). We observed that this builder order is more efficient in sampling the hairpin under positional and hydrogen bonding restraints, than the simple sequential order.

**Figure 13:**
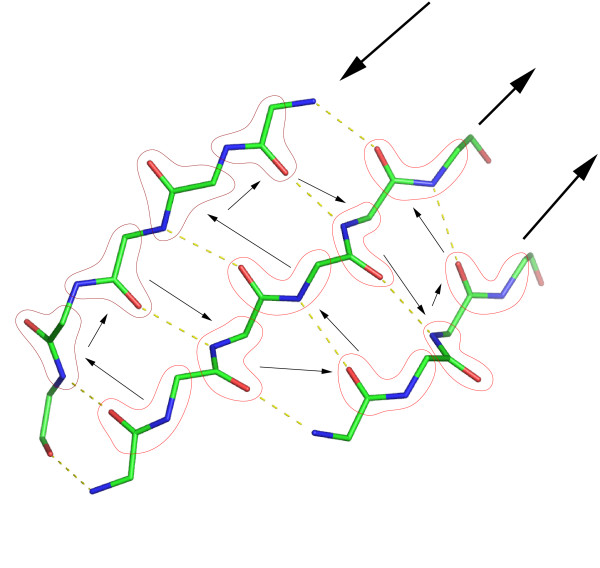
*β *sheet building by identifying the ladder and sampling along the steps. Multiple strands and both (anti/parallel) arrangements can be sampled within hydrogen bonding (angle C-O-N and distance O-N) restraints.

*Rapper***tk **extends this scheme of sampling *β*-sheets to parallel sheets and arbitrarily many strands (see Fig. 13). If residue positions (...*i *- 1, *i*, *i *+ 1...) correspond to positions (...*j *- 1, *j*, *j *+ 1...) in parallel *β*-sheets, hydrogen bonding distance restraints are applied on (*N*^*i*^, *O*^*j*-1^), (*O*^*i*^, *N*^*j*+1^), (*N*^*i*+2^, *O*^*j*+1^), (*O*^*i*+2^, *N*^*j*+3^) etc. In antiparallel sheets, where residue positions (...*i *- 1, *i*, *i *+ 1...) correspond to positions (...*j *+ 1, *j*, *j *- 1...), distance restraints are applied on (*N*^*i*^, *O*^*j*^), (*O*^*i*^, *N*^*j*^), (*N*^*i*+2^, *O*^*j*-2^), (*O*^*i*+2^, *N*^*j*-2^) etc. Sheets with multiple strands are tricky due to the variable number of hydrogen bonds between adjacent strands. Additionally, a strand may be linked to other strands in both parallel and antiparallel arrangements, e.g. in a 3-stranded sheet with corresponding residue positions (...*i *- 1, *i*, *i *+ 1...), (...*j *- 1, *j*, *j *+ 1...) and (...*k *+ 1, *j*, *j *- 1...), residue *j *is involved in (*N*^*j*^, *O*^*i*-1^), (*O*^*j*^, *N*^*i*+1^) while residue *j *+ 1 forms hydrogen bonds (*N*^*j*+1^, *O*^*k*-1^), (*O*^*j*+1^, *N*^*k*-1^); this pattern repeats every alternate residue. This scheme is used in the next example.

#### 3.2.4 All-atom model generation from approximate secondary structure information and particle shape

Techniques like EM and SAXS are valued for their ability to estimate macromolecular shape and to help in global relative positioning of parts of the particle. Automatic identification of secondary structures and prediction of their topology is possible [25,26] by morphological analysis of EM data. Coupled with secondary structure prediction from sequence, this generates approximate positional restraints on *C*_*α *_atoms in secondary structures. We demonstrate here that *Rapper***tk **can combine the shape and secondary structure positional restraints to generate atomic models.

**Figure 14:**
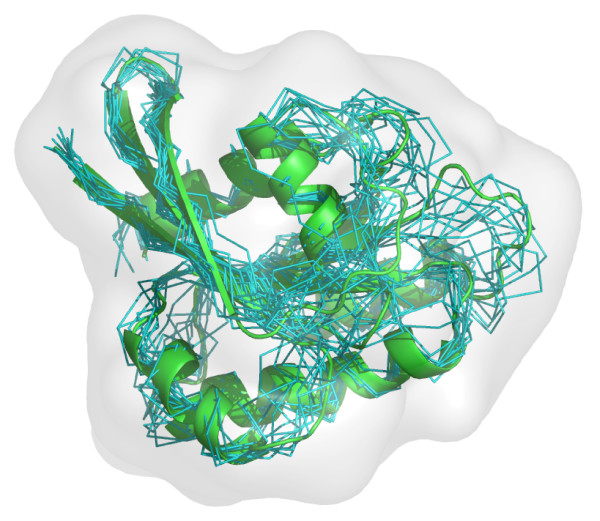
Combining shape and secondary structure skeleton to generate atomic models.

In order to simulate this scenario, we generated an artificially blurred electron density map at 10Å resolution using EMAN [27] and built into the envelope defined by 1 *σ *contour. 3Å *C*_*α *_positional restraints are placed on residues in secondary structures. There are no positional restraints on sidechains and loops. Hydrogen bonding restraints are used for *β *sheets as described earlier, and also on *α *helices. Ten models thus generated are shown in Fig. 14. Model variations are large in loops but not in secondary structures due to secondary-structure-specific sampling style used by *Rapper***tk**.

## 4 Discussion and Conclusions

*Rapper***tk**'s design makes it possible to apply discrete restraint-based modeling to a variety of problems robustly and easily because

• Introducing new builders, restraints, samplers and search strategies is easy.

• Any level of granularity can be chosen to represent the structure.

• Automatic ordering of builders and restraints spares the user from the tedious task, but a preferred order may be imposed if needed.

• Any number of coordinates may be known before modelling. They can be used as restraints or to make seed builders or just as steric obstructions.

• Ensemble building and average restraints can be introduced easily by adding restraints which check the average value of some property of the conformational pool.

The modularity and flexibility of *Rapper***tk **makes it an attractive platform for carrying out discrete restraint-based modeling tasks under a variety of restraints, as we have demonstrated here. *Rapper***tk **can also be useful to generate decoy sets useful in developing energy functions for discriminating between non-native and native conformations.

Our immediate goals with this toolkit include exploring protein-ligand and protein-RNA interface conformations, aiding automation of X-ray refinement and developing a protocol for interpreting NMR restraints. To address these tasks more effectively, some more features will likely be needed. For instance, non-binary restraints are not at present implemented. To introduce such analog restraints, the population seach strategy will be modified to allow scoring of conformational extensions as well as members of an ensemble of conformations. We also intend to implement coarse samplers to address sparse restraint scenarios, e.g. by analyzing geometric preferences between adjacent secondary structure elements, a coarse-grained secondary structure incremental sampling can be achieved. Another concern is that although builder order in *Rapper***tk **is flexible, still it is a linear order, hence concerted conformational change is not possible. We are working on implementing a strategy inspired by the SCWRL algorithm [28], which will operate at the level of side-chains as well as fragments and optimize the conformational possibilities independent of builder order. Another strategy under consideration involves simulated annealing and incorporation of conformation-modifiers which tweak the structure in a particular way, e.g. local backbone moves, rigid-body fragment movements, sidechain flips and so on. Tweakers will form the move-set for simulated annealing which will be used to obtain a coarse structural framework that will be further explored to get atomic models.

In conclusion, we believe that *Rapper***tk **will prove to be a useful platform for conformational sampling and searching for a wide range of applications.

## Availability and requirements

• Project name: *Rapper***tk**

• Project home page: 

• Operating system(s): Linux

• Programming language: Python C++

• Other requirements: swig

• License: GNU GPL

• Academic users can download the source code project home page and also as additional file with this paper. Commercial users should contact the authors for a license.

## Abbreviations

**GABB **Genetic algorithm with branch-and-bound algorithm

**PDB **Protein Data Bank

**DFS **Depth-first search

**SWIG **Simplified Wrapper and Interface Generator

**RMSD **Root mean square deviation

## Authors' contributions

SPG designed and implemented the software library and drafted the manuscript. AMK performed benchmarking runs and contributed the corresponding section in the manuscript. TLB critically reviewed the manuscript and provided valuable guidance. All authors read and approved the final manuscript.

## Figures and Tables

**Table 1:** Set of target proteins

Pdb Id^a^	Protein Name	Resolution^b^	Size^c^
1cem	Cellulase	1.65	363
1nif	Nitrite reductase	1.6	333
1php	Phosphoglycerate kinase	1.65	394
3app	Penicillopepsin	1.8	323
3pte	Transpeptidase	1.6	347
4enl	Enolase	1.9	436
5cpa	Hydrolase (c-terminal peptidase)	1.54	307
8abp	Arabinose binding protein	1.49	305
8tln:E	Thermolysin	1.60	316

^a^PDB code (and chain identifier, if necessary)

^b^Resolution of the crystal structure in Å.

^c^Number of amino acids in the protein chain.

**Table 2:** Comparison of mainchain RMSD under l.0Å *C*_*α *_restraint.

	RAPPER		*Rapper***tk **Forward		*Rapper***tk **Backward	
Target	Mainchain^*a*^	*C*_*α*_^*b*^	Mainchain^*a*^	*C*_*α*_^*b*^	Mainchain^*a*^	*C*_*α*_^*b*^

1cem	0.69 (0.01)	0.66 (0.01)	0.69 (0.01)	0.67 (0.01)	0.68 (0.01)	0.66 (0.01)
1nif	0.72 (0.01)	0.67 (0.01)	0.71 (0.01)	0.67 (0.01)	0.72 (0.02)	0.67 (0.02)
1php	0.70 (0.01)	0.66 (0.01)	0.70 (0.01)	0.66 (0.01)	0.70 (0.01)	0.66 (0.01)
3app	0.71 (0.02)	0.67 (0.01)	0.72 (0.02)	0.67 (0.01)	0.70 (0.01)	0.66 (0.01)
3pte	0.71 (0.01)	0.67 (0.01)	0.71 (0.01)	0.67 (0.01)	0.71 (0.01)	0.67 (0.01)
4enl	0.72 (0.02)	0.68 (0.01)	0.71 (0.02)	0.67 (0.02)	0.71 (0.01)	0.67 (0.01)
5cpa	0.73 (0.02)	0.68 (0.02)	0.73 (0.02)	0.67(0.01)	0.72 (0.02)	0.67 (0.02)
8abp	0.71 (0.01)	0.67 (0.01)	0.72 (0.02)	0.67(0.02)	0.71 (0.01)	0.67 (0.01)
8tn:E	0.71 (0.02)	0.67 (0.01)	0.70 (0.02)	0.67(0.02)	0.71 (0.01)	0.67 (0.01)

^*a *^Ensemble average main chain RMSD.

^*b *^Ensemble average *C*_*α *_RMSD.

**Table 3:** Comparison of model accuracy under different *C*_*α *_restraint thresholds

		*C*_*α *_restraint threshold in Å			
		0.5	1	1.5	2
RAPPER ^*c*^	MC^*b*^	0.42 (0.01)	0.71 (0.01)	1.00 (0.02)	1.30 (0.03)
	*C*_*α*_^a^	0.35 (0.01)	0.67 (0.01)	0.98 (0.02)	1.29 (0.02)
*Rapper***tk **Forward^*c*^	MC^*b*^	0.43 (0.01)	0.71 (0.01)	1.00 (0.02)	1.30 (0.03)
	*C*_*α*_^a^	0.35 (0.01)	0.67 (0.01)	0.98 (0.02)	1.29 (0.03)
*Rapper***tk **Backward^*d*^	MC^b^	0.42 (0.01)	0.71 (0.01)	1.00 (0.02)	1.30 (0.03)
	*C*_*α *_^a^	0.35 (0.01)	0.67 (0.01)	0.98 (0.02)	1.29 (0.03)

Values in parentheses indicate standard deviations.

^a^Ensemble average *C*_*α *_RMSD [Å] over all successfully modelled proteins.

^b^Ensemble average main chain RMSD [Å] over all successfully modelled proteins.

^c^No models could be generated for targets 4ENL, 8ABP, 8TLN under 0.5Å *C*_*α *_restraint by RAPPER and *Rapper***tk **forward building.

^d ^No models could be generated for targets 3PTE, 4ENL, 5CPA, 8ABP under 0.5Å restraint by *Rapper***tk **backward building

**Table 4:** Comparison of accuracy of all atom modelling

	RAPPER				*Rapper***tk**				*Rapper***tk **Guided			
PDB ID	MC^*a*^	AA^*b*^	*χ*_1_^*c*^	*χ*_1,2_^*d*^	MC^*a*^	AA^*b*^	*χ*_1_^*c*^	*χ*_1,2_^*d*^	MC^*a*^	AA^*b*^	*χ*_1_^*c*^	*χ*_1,2_^*d*^
1cem	0.69	1.06	38.6	36.5	0.69	1.06	38.9	37.2	0.61	0.97	38.7	37.0
1nif	0.71	1.10	40.4	42.7	0.71	1.09	39.6	42.4	0.68	1.04	39.8	42.5
1php	0.7	1.07	39.4	41.1	0.70	1.07	40.1	41.4	0.63	0.99	39.8	41.9
3app	0.71	1.08	46.1	53.1	0.71	1.08	46.4	53.4	0.66	1.00	44.3	52.1
3pte	0.71	1.07	44.0	39.7	0.71	1.07	42.8	39.8	0.65	0.98	43.1	38.9
4enl	0.71	1.06	42.2	39.4	0.71	1.06	42.4	39.3	0.64	0.98	41.9	39.4
5cpa	0.73	1.13	44.74	48.3	0.72	1.12	43.6	48.1	0.65	1.04	42.9	47.6
8abp	0.71	1.10	41.6	41.9	0.72	1.10	40.5	41.7	0.63	1.01	41.2	41.3
8tln:E	0.71	1.07	43.5	39.1	0.7	1.06	43.4	38.6	0.64	0.98	42.3	38.1

^*a *^Average main chain RMSD (Å) of 50 models for each protein, averaged over all proteins in target set.

^*b *^Average all atom RMSD (Å) of 50 models for each protein, averaged over all proteins in target set.

^*c *^Percentage of side chains with *χ*_1 _> 40° of the equivalent *χ*_1 _in the crystal structure, averaged over all proteins in target set.

^*d *^Percentage of side chains with *χ*_1,2 _> 40° of the equivalent *χ*_1,2 _in the crystal structure, averaged over all proteins in target set.
